# Clinical and gonadal features and early surgical management of 45,X/46,XY and 45,X/47,XYY chromosomal mosaicism presenting with genital anomalies

**DOI:** 10.1016/j.jpurol.2011.12.012

**Published:** 2013-04

**Authors:** M.K. Farrugia, N.J. Sebire, J.C. Achermann, A. Eisawi, P.G. Duffy, I. Mushtaq

**Affiliations:** a Department of Urology, Great Ormond Street Hospital for Children, Great Ormond Street, London WC1N 3JH, UK; b Department of Pathology, Great Ormond Street Hospital for Children, Great Ormond Street, London WC1N 3JH, UK; c Developmental Endocrinology Research Group, UCL Institute of Child Health, 30 Guilford Street, London WC1N 1EH, UK

**Keywords:** 45,X/46,XY, 45,X/47,XYY, Mixed gonadal dysgenesis, Chromosomal mosaicism, Ambiguous genitalia, Disorder of sex development, Streak gonad, Dysgenetic gonad

## Abstract

**Objective:**

The 45,X/46,XY and 45,X/47,XYY group of patients includes some of those previously diagnosed with ‘mixed gonadal dysgenesis’. Our aim was to establish the clinical and gonadal spectrum, and early surgical management, of patients with chromosomal mosaicism presenting with genital anomalies.

**Patients and methods:**

We performed a retrospective review of patients with 45,X/46,XY or 45,X/47,XYY mosaicism presenting with genital ambiguity between 1988 and 2009. At least one gonadal biopsy or gonadectomy specimen was available for each patient. Gonadal histology was re-evaluated by a paediatric pathologist.

**Results:**

Of 31 patients with 45,X/46,XY (*n* = 28) or 45,X/47,XYY (*n* = 3) mosaicism and genital anomalies, 19 (61%) were raised male. Histology of 46 gonads was available from patients who had undergone a gonadectomy or gonadal biopsy, at a median age of 9.5 months. 18 gonads were palpable at presentation, including 5 (28%) histologically unremarkable testes, 2 streak gonads, and 1 dysgenetic gonad with distinct areas of testicular and ovarian stroma but no oocytes. All intra-abdominal gonads were found to be dysgenetic testes (of which 2 were noted to have pre-malignant changes) or streaks, apart from 1 histologically unremarkable testis. 15 (48%) patients had other anomalies, most commonly cardiac and renal; 4 (13%) had a Turner phenotype.

**Conclusion:**

The anatomy and gonadal histology of 45,X/46,XY and 45,X/47,XYY individuals with genital ambiguity do not conform to a set pattern, and hence management of each patient should be individualized according to detailed anatomical and histological assessment.

## Introduction

The 2006 consensus statement on the management of disorders of sex development (DSD) introduced the concept of a DSD classification based on karyotype. This consensus included ‘45,X/46,XY and 45,X/47,XYY (mixed gonadal dysgenesis, ovotesticular DSD)’ patients in the sex chromosome DSD group [1]. Prior to the consensus statement, patients with 45,X/46,XY and 45,X/47,XYY mosaicism presenting with genital anomalies would have been diagnosed with ‘mixed gonadal dysgenesis’ (MGD). However, published case series of MGD have included patients with 45,X/46,XY and 45,X/47,XYY karyotypes, as well as 46,XY [2–4]. The latter group of patients now form part of the 46,XY DSD group in the consensus classification. The aim of our study was to review the clinical and gonadal features and early surgical management of patients with 45,X/46,XY and 45,X/47,XYY chromosomal mosaicism presenting with genital anomalies.

## Patients and methods

We performed a retrospective study of a cohort of patients presenting with genital ambiguity and found to have a 45,X/46,XY or 45,X/47,XYY karyotype, managed in the Great Ormond Street Hospital for Children between 1988 and 2009. At least one gonadal biopsy or gonadectomy specimen was available for each patient. Each specimen was reviewed and re-classified by a specialist paediatric pathologist (NJS), according to previously published criteria [5]. Definitions are summarized in Table 1. In all cases presence of morphological intratubular germ cell neoplasia was specifically searched for. Data were expressed as median (range).

Our current practice is for all newly referred patients with a possible DSD to be initially assessed and investigated by the endocrine team. Patients are also examined at the same time by a paediatric urologist and input from a clinical psychologist is provided in most cases [6]. Clinical findings and investigation results are then discussed at a multidisciplinary meeting involving endocrinologists, paediatric urologists, geneticists, adolescent urologists and gynaecologists, and clinical psychologists. Management plans and the potential sex of rearing are discussed with the family at all stages of this process. Patients are typically re-admitted for an examination under anaesthetic, cystoscopy and laparoscopy at 2-3 months of age, although in cases where the findings may have a bearing on the sex of rearing, the procedures may need to be performed more urgently, in the first few weeks of life. (Early in the series, laparoscopy was not generally available and hence some of the patients underwent a laparotomy.) In patients raised male, intra-abdominal gonads are biopsied and a laparoscopic first-stage Fowler-Stephens orchidopexy is performed if indicated. Obvious streak gonads are removed laparoscopically. In patients raised male with palpable but undescended gonads, an orchidopexy is performed before 1 year of age. To date, the gonad has not been routinely biopsied, unless it appeared to be abnormal macroscopically. Intrascrotal gonads are not normally biopsied. Hypospadias repair is performed at 1-2 years of age. In patients raised female, both gonads are typically removed. Appropriateness and timing of reconstructive surgery in female patients depends on a number of factors. All gonads excised or biopsied are routinely submitted for histological evaluation. Specimens were assessed for standard morphological features of intratubular germ cell neoplasia with placental-like alkaline phosphatase (PLAP) immunostaining on all cases with seminiferous tubules present, using standard avidin-biotin procedure on a diagnostic automated LEICA bond immunostainer (Novocastra PLAP clone 8A9).

## Results

A database search yielded a total of 31 patients with genital anomalies and chromosomal mosaicism, for whom full clinical and pathological information was available. Karyotype was 45,X/46,XY in 28 individuals and 45,X/47,XYY in 3 individuals. All children were referred to the endocrine team with genital anomalies shortly after birth, except for 4 patients, of whom 3 were referred from other centres within the first year of life (Australia, Qatar, Scotland) and one was diagnosed at 4 years of age following a referral for hypospadias repair. Only 1 set of parents were known to be consanguineous. None of the cases included in this series were prenatally diagnosed, although in two cases there was a prenatal suspicion of Turner syndrome. Median gestational age at birth was 39 (27–41) weeks.

Nineteen patients were raised male (*n* = 18 45,X/46,XY and *n* = 1 45,X/47,XYY). Their clinical features at presentation are summarized in Table 2A. Median age at initial surgical intervention in this group of patients was 19.5 (0.5–72) months, and the interventions were as follows: laparoscopy/laparotomy (*n* = 11), gonadectomy (*n* = 11, including 1 bilateral gonadectomy), staged hypospadias repair (*n* = 18), orchidopexy (*n* = 9). The utriculus was excised in 2 patients, and 1 patient had a vaginectomy. Median age at gonadal biopsy or gonadectomy was 6.5 (1–27) months. Twelve patients were raised female (*n* = 10 45,X/46,XY and *n* = 2 45,X/47,XYY), and their clinical features at presentation are summarized in Table 2B. Median age at initial surgical intervention was 12.0 (1–180) months, and the interventions were as follows: laparoscopy/laparotomy (*n* = 10), bilateral gonadectomies (*n* = 12), genitoplasty (*n* = 7) and clitoral reduction (*n* = 5).

**Figure 1:**
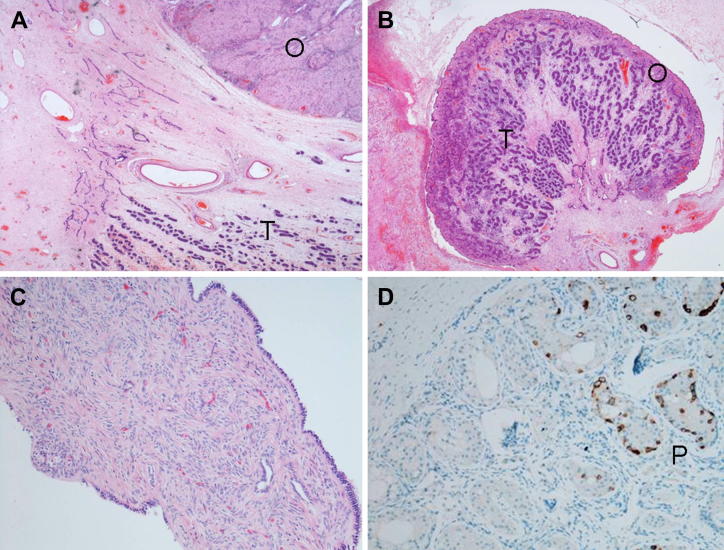
A: An unusual dysgenetic gonad showing distinct areas of ovarian-type stroma (O) and testicular (T) tissue, but no ovarian follicles or germ cells. B: Dysgenetic testis showing disorganized testicular tissue (T) surrounded by atypical ovarian-type stroma (O). C: Streak gonad lacking germ cells. D: Placental-like alkaline phosphatase immunostaining, seen as brown-staining (P), suggestive of delayed maturation or intratubular germ cell neoplasia unclassified.

The histology of 46 gonads was available from patients who had undergone a gonadectomy or gonadal biopsy. Median age at gonadal biopsy or gonadectomy was 9.5 (1–180) months. Gonadal histology is summarized in Table 3 and illustrated in Fig. 1A–D. Of the total cohort (*n* = 31), 7 patients (23%) had bilateral palpable gonads (inguinal or scrotal). Seventeen patients (55%) had a palpable gonad on one side, and an impalpable gonad on the contralateral side. Of the palpable gonads, 16 (52%) were in an inguinal location, 10 (32%) were scrotal, and in 5 (16%) the exact location was unknown. Histology of the palpable gonad was only available for those gonads that were biopsied or removed. Of note, 3 gonads palpable in the inguinal region were found to be streak gonads (*n* = 2) and an unusual dysgenetic gonad (Fig. 1A). The latter gonad exhibited distinct, polar areas of testicular tissue and ovarian-type stroma with cysts reminiscent of follicular cysts but no oocytes or normal ovarian tissue. This appearance differed from the structure of the other dysgenetic gonads in our series, where the ovarian-type stroma was at the periphery of the gonad surrounding the testicular tissue (Fig. 1B). The histology of the scrotal gonad was available in 3 out of 10 cases, of which only 1 was a histologically unremarkable testis, the remaining 2 being a dysgenetic testis and a streak gonad (Fig. 1C). The normal testis was removed from a patient reared female, whereas the dysgenetic testis and streak gonad were from patients reared male, and were initially palpably indistinguishable from a normal testicle. Hence, they were not biopsied in infancy. However, both gonads ‘ascended’ later in childhood, were subsequently noted to be abnormal at orchidopexy, and were biopsied. Overall, histology of the palpable gonad was known in 18 cases, of which 5 (28%) were histologically unremarkable testes.

Seven patients (23%) presented with bilateral impalpable gonads. In 2 cases, an intra-abdominal gonad could not be found suggesting agenesis or regression. The majority of intra-abdominal gonads identified in the entire series were either dysplastic testes (38%) or streaks (45%). Only 1 intra-abdominal testis (3%) was histologically unremarkable. Only 1 patient had a gonadectomy after 30 months of age. This patient (45,X/47,XYY raised female) had emigrated to the Middle-East soon after diagnosis in infancy, and re-presented with a pair of intra-abdominal gonads still in situ at 15 years of age. Bilateral gonadectomy was performed, and histology revealed bilateral dysgenetic testes containing numerous morphologically normal but PLAP-positive cells (suggestive of dysmaturity or early intratubular germ cell neoplasia unclassified; Fig. 1D). The patient, who was raised female, also had features of Turner syndrome. There were no cases with morphological features of intratubular germ cell neoplasia despite the 1 case with immunohistochemical staining features described above, and no cases of gonadoblastoma or frank malignancy.

Fifteen patients (48%) were found to have other anomalies. The patient with 45,X/47,XYY karyotype and bilateral PLAP-positive gonads also had total anomalous pulmonary venous drainage, a high arched palate, skin pigmentation, long fingers, deafness, and reduced vision. Four further patients had cardiovascular anomalies (ventricular septal defect, aortic stenosis, aortic regurgitation and patent ductus arteriosus with patent foramen ovale), giving a 16% incidence of documented cardiovascular anomalies in this series. Five patients (16%) were found to have renal abnormalities including a horseshoe kidney in 3 patients, and 1 duplex and 1 multicystic dysplastic kidney. One of the patients with a horseshoe kidney also had bilateral colobomas and reduced vision. Only 1 patient had both renal and cardiac anomalies. Two patients were noted to have adrenocortical rests on their testes, of whom 1 had clinical adrenocortical insufficiency. Four patients (13%) were noted to have significantly short stature and curved fingernails, compatible with a Turner phenotype. Two patients had gastrointestinal problems (pyloric stenosis and coeliac disease).

## Discussion

45X/46,XY and 45,X/47,XYY chromosomal mosaicism is rare, with an incidence of 1.7/10,000 pregnancies. Only 5–10% of individuals with a prenatally diagnosed mosaic karyotype of 45,X/46,XY will have ambiguous genitalia at birth, with the remainder being phenotypic male [7]. Chang et al. reported a series of 92 prenatally diagnosed cases of chromosomal mosaicism, including 88 45,X/46,XY and 4 45,X/47,XYY mosaics, of whom only 4 (5%) were noted to have genital anomalies. There was no relation between the percent mosaicism (the number of 45,X cells relative to the total number of cells) and the presence or degree of abnormalities. Long-term follow-up revealed that mental status and stature were normal, except for 1 male whose height was below the fifth percentile [7]. There appears to be a phenotypic difference between prenatally and postnatally diagnosed cases of 45,X/46,XY or 45,X/47,XYY mosaicism, the latter being more likely to present with genital anomalies [8,9]. Although the presentation of 45,X/46,XY mosaicism with genital anomalies only represents a small proportion of cases with this karyotype, these are the most challenging cases to manage. The genital anomalies reported to date range from phenotypic males with cryptorchidism or hypospadias to phenotypic females with gonadal dysgenesis [10]. The more typical combination of a unilateral testis, contralateral streak gonad and persistent Müllerian derivatives was previously called ‘mixed gonadal dysgenesis’ (MGD), a common diagnosis given to 45,X/46,XY mosaic infants with genital anomalies [11,12]. However, using this term as a diagnosis can be confusing, as most published series of MGD have included patients with 46,XY as well as mosaic karyotypes [2–5,14]. The consensus statement has since re-classified patients with 45,X/46,XY mosaicism and genital anomalies (sex chromosome DSD) and 46,XY patients with ‘partial gonadal dysgenesis’ (46,XY DSD) into two separate categories [1].

Our series revealed that 45,X/46,XY mosaicism and its variants may be associated with other anomalies, namely cardiac (16%) and renal (16%). Only 4 patients (13%), however, had other Turner features such as short stature and curved fingernails. Although this combination of features is typical of Turner syndrome (most commonly 45,X or 45,X/46,XX mosaic) [13], there are only limited reports of their association with 45,X/46,XY mosaicism [8,14]. Wallace and Levin also documented a high incidence of cardiac anomalies in 45,X/46,XY mosaics, including bicuspid aortic valve, retro-oesophageal aortic arch, aberrant left subclavian artery, coarctation and dissection [14]. Cardiac and renal imaging is therefore recommended in these infants at diagnosis.

Sex assignment may be challenging, and the surgical approach has evolved over the years. Papers published only 20–30 years ago recommended early gonadectomy and a female sex assignment [2–4], an approach which has recently changed, with increased emphasis on a conservative approach and the delay of irreversible surgery until adulthood. Several factors are involved in decision making regarding sex assignment, including long-term likely gender identity; urological and sexual function and genital appearance; the possible need for endocrine replacement therapy to induce puberty and throughout adult life; and any capacity for future fertility. All these factors are in addition to the malignancy risk associated with the condition, which has made gonadal management, particularly in patients raised male, increasingly challenging. The fact that the prevalence of malignant germ cell tumours is increased in patients with DSD containing Y chromosome material in their karyotype is well documented, and is probably related to the presence and aberrant expression of the testis-specific protein on Y (TSPY) gene, proximal on Yp [15–20]. The ectopic position of the dysgenetic testis adds to this risk because the prevalence of germ cell tumours in simple cryptorchidism is estimated at 4–10 times the normal prevalence of 6–11 per 100,000 [21]. Cools et al. reported that in series of patients with MGD the overall prevalence of germ cell tumours is 18 out of 119 patients (15%) [18]. The same group went on to specifically look at tumour risk in relation to clinical characteristics, in a series of 48 patients with 45,X/46,XY mosaicism [22]. It is interesting to note that the authors also describe the occurrence of 1 intra-abdominal gonad containing marked ovarian differentiation with ovarian follicles in their series, which is similar to the finding in our study. An inguinal streak gonad was also encountered, although all scrotal gonads were found to be testes. Fifteen gonads, including 4 with in-situ neoplastic lesions, in 12 different patients, were found to have pre-malignant characteristics. Tumour risk was significantly reflected by clinical phenotype, and revealed to be very high (52%) in patients with an ambiguous phenotype. Inguinal gonads displayed (pre)malignant characteristics more frequently compared with scrotal or abdominal gonads; however the difference was not statistically significant. Conversely, the phenotypically female patients were found to have a lower tumour risk, which the authors attribute to the fact that these patients were more likely to have streak or ‘vanished’ gonads. This finding is of course in contrast to that in our series, whereby the only pre-malignant gonads were found in a phenotypically female patient with intra-abdominal dysgenetic testes.

The long-term potential for hormone production and fertility in mosaic individuals is not clear in the literature. Three of 27 patients in the Telvi series went through a normal puberty even after their unilateral streak gonads were removed [8]. Robboy et al. reported that even when MGD gonads in 45,X/46,XY individuals were shown to be rich in germ cells when biopsied in infancy, gonadectomy 6–13 years later demonstrated the same gonads to be densely hyalinized and fibrous [4]. There are also several reports of phenotypic males with 45,X/46,XY mosaicism presenting to infertility clinics and being found to be azoospermic [23–25].

Our study is limited by its retrospective nature and small patient numbers. The underlying genetics and gonadal pathology in this rare condition are currently being elucidated in greater detail than ever before [26,27], utilizing immunohistochemical techniques that were not previously available. Our gonadal specimens were not tested for aberrant TSPY expression or other genetic markers, such as the octamer binding transcription factor 3/4 (OCT3/4) and c-KIT ligand stem cell factor, which have been successfully used in recent studies [22]. Cools et al. hypothesized that the combination of prolonged OCT3/4 expression and TSPY expression in the germ cells of DSD patients is of pathogenetic relevance for the development of gonadoblastoma in these patients [28]. Widespread availability of these techniques may become of great value when assessing the tumour risk of dysgenetic testes biopsied at orchidopexy or post puberty, as advised by the consensus statement [1], and we feel that this would be the best way forward when managing patients raised male, whose gonads are preserved. In addition, since our centre is a paediatric one, we do not currently know the adult outcome of some of our post-pubertal patients, whose care has been transferred to an adolescent uro-gynaecology centre. We therefore aim to trace these patients and study their endocrine and pubertal outcome in a separate study.

## Conclusion

The anatomy and gonadal histology of 45,X/46,XY and 45,X/47,XYY individuals with genital abnormalities is more complex than previously thought, and our study highlights the importance of detailed anatomical and histological assessment at an early age. In patients raised male, where dysgenetic testes are retained, biopsy at orchidopexy and post puberty with appropriate immuo-histochemical staining is warranted to identify patients at increased risk of malignancy.

## Funding source

None. One author (JCA) is a Wellcome Trust Senior Fellow in Clinical Science (079666).

## Conflict of interest

The authors do not have any disclosures or conflicts of interest.

## Figures and Tables

**Table 1:** Gonadal types and histological definitions.

Gonad	Definition
‘Unremarkable’ testis	With or without secondary non-specific cryptorchid-associated changes
Ovary	Normal architecture with typical ovarian stroma and oocytes
Dysgenetic/dysplastic gonad	Testicular features with abnormal architecture and ovarian-type gonadal stroma
Streak gonad	Abnormal architecture and ovarian-type stroma only, with absence of germ cells or other specific features
Ovotestis	Clear separate areas of definite testicular and ovarian tissue (including ovarian follicles/oocytes)

**Table 2:** A and B. Clinical characteristics of patients with 45,X/46,XY and 45,X/47,XYY presenting with genital anomalies.

A) Clinical features of patients raised male (*n* = 19)			
Phallus	Penoscrotal/perineal hypospadias (*n* = 18)	Normally placed terminal meatus (*n* = 1)	
Scrotum	Normal scrotum (*n* = 13)	Bifid scrotum (*n* = 3)	Hemiscrotum (*n* = 3)
Gonads	Palpable gonads bilaterally (*n* = 5)	One palpable gonad (*n* = 13)	No palpable gonads (*n* = 1)
Müllerian structures	Utriculus (*n* = 11)	Hemi-utriculus (*n* = 1)	No Müllerian structures (*n* = 7)
B) Clinical features of patients raised female (*n* = 12)			
Phallus	Clitoromegaly (*n* = 12)		
Gonads	Palpable gonads bilaterally (*n* = 2)	One palpable gonad (*n* = 5)	No palpable gonads (*n* = 5)
Müllerian structures	Utriculus (*n* = 7)	Hemi-utriculus (*n* = 3)	No Müllerian structures (*n* = 2)

**Table 3:** Summary of gonadal histology.

Palpable (*n* = 31)			Impalpable (*n* = 31)	
Inguinal (*n* = 16) 5 not biopsied 4 dysgenetic testes 3 normal testes 2 streak gonads 1 immature testis 1 ovotestis-like gonad	Scrotal (*n* = 10) 7 not biopsied 1 dysgenetic testis^a^ 1 normal testis 1 streak gonad^a^	Unknown (*n* = 5) 1 not biopsied 2 dysgenetic testes 1 immature testis 1 normal testis	Absent (*n* = 2)	Intra-abdominal (*n* = 29) 13 streak gonads 11 dysgenetic testes (2 PLAP-positive^b^) 3 immature testes 1 normal testis 1 unknown

a Gonads initially scrotal but ‘ascended’ and were biopsied at orchidopexy.

b Both placental-like alkaline phosphatase positive testes were found in the same patient.
